# Role-Playing Computer Ethics: Designing and Evaluating the Privacy by Design (PbD) Simulation

**DOI:** 10.1007/s11948-020-00250-0

**Published:** 2020-07-01

**Authors:** Katie Shilton, Donal Heidenblad, Adam Porter, Susan Winter, Mary Kendig

**Affiliations:** 1 College of Information Studies, University of Maryland College Park, College Park, MD USA; 2 Department of Computer Science, University of Maryland College Park, College Park, MD USA; 3 DELTA Resources, Inc, Washington, DC USA

**Keywords:** Ethics simulation, Ethics education, Computer ethics, Values at play

## Abstract

There is growing consensus that teaching computer ethics is important, but there is little consensus on how to do so. One unmet challenge is increasing the capacity of computing students to make decisions about the ethical challenges embedded in their technical work. This paper reports on the design, testing, and evaluation of an educational simulation to meet this challenge. The privacy by design simulation enables more relevant and effective computer ethics education by letting students experience and make decisions about common ethical challenges encountered in real-world work environments. This paper describes the process of incorporating empirical observations of ethical questions in computing into an online simulation and an in-person board game. We employed the *Values at Play* framework to transform empirical observations of design into a playable educational experience. First, we conducted qualitative research to discover when and how values levers—practices that encourage values discussions during technology development—occur during the design of new mobile applications. We then translated these findings into gameplay elements, including the goals, roles, and elements of surprise incorporated into a simulation. We ran the online simulation in five undergraduate computer and information science classes. Based on this experience, we created a more accessible board game, which we tested in two undergraduate classes and two professional workshops. We evaluated the effectiveness of both the online simulation and the board game using two methods: a pre/post-test of moral sensitivity based on the Defining Issues Test, and a questionnaire evaluating student experience. We found that converting real-world ethical challenges into a playable simulation increased student’s reported interest in ethical issues in technology, and that students identified the role-playing activity as relevant to their technical coursework. This demonstrates that roleplaying can emphasize ethical decision-making as a relevant component of technical work.

## Introduction

The software industry is facing a crisis of ethics (Vallor 2016; Wachter-Boettcher 2017). The public is increasingly aware of the amount of personal data collected by applications and platforms, and the troubling ends to which this data has sometimes been put, including encouraging addiction (Lewis 2017), enabling discriminatory profiling (O’Neil 2017; Sweeney 2013), mass manipulation of public discourse, and election interference (Rosenberg et al. 2018). Teaching the software engineers who curate sensitive and personal data to make wise ethical decisions is thus a critical educational challenge. Although some accredited computer science programs are required to cultivate “an understanding of professional, ethical, legal, security and social issues and responsibilities” (ABET Computing Accreditation Commission 2017), the ethics crisis demonstrates that current approaches do not provide students with the skills to successfully navigate complex ethical issues in the real world.

The reasons for this failure are complex. The social impact of computing has been included in the curriculum recommendations made by the Association for Computing Machinery (ACM) as far back as 1978 (ACM Committee on Professional Ethics 2018). Two major professional organizations, the ACM and the Institute of Electrical and Electronics Engineers (IEEE), began accrediting computer science programs in 1991, and accreditation requirements included coursework in the areas of “social, ethical, and professional issues” (Tucker 1991). A 2001 update to the curriculum requirements recommended that ethics be taught throughout the computing curricula (The Joint Task Force on Computing Curricula 2001). Recently, there has been renewed high-profile interest in computer ethics education (Singer 2018), and large numbers of academics are teaching ethics courses in computer science, information science, and human–computer interaction.^1^

However, scholars have critiqued the ways that ethics are taught in computing as too limited in both integration and depth, focusing on awareness and knowledge rather than practice or action. Donald MacKenzie and Judy Wajcman wrote in 1999 that “the teaching of engineering does not, in our experience, generally reflect the technologists’ awareness of the intertwining of the ‘social’ and ‘technical’ in engineering practice” (MacKenzie and Wajcman 1999, pp. 15–16). More recently, longtime computer ethics educators Charles Huff and Almut Furchert diagnosed a specific problem of existing computing pedagogy:

Alongside knowledge of the good, we need to gain practical wisdom (phronesis) that guides our ethical action. … Herein lies an uncomfortable tension: While the ethics code is full of the obligation to design systems with ethical concern in mind, the typical computing ethics textbook does not help one learn how to do that (2014, p. 26).

Teaching wise practice, rather than just knowledge, is a difficult challenge. Case studies are a staple of computer ethics instruction that are used as prompts for discussion or critical writing that facilitates conceptual mastery (Aiken 1983; Parker 1979; Harmon and Huff 2000). However, case studies tend to highlight anti-exemplars and demonstrate paths to avoid rather than paths to follow, limiting their value as exemplars of good practice. The end result of case studies is also known, providing the clarity of hindsight, where in the moment, there may have been questions and ethical uncertainty.

Beyond teaching wise practice, computer ethics education must convince students that it is relevant to their professional aspirations. For example, Cech (2014) has illustrated that U.S. engineering students often become *less* engaged with social issues over their time in college or university programs. Cech faults prevalent ideologies that teach that engineering work is morally neutral and apolitical; that “softer” social issues are distinct from “harder” technical issues; and finally, that meritocracy ensures that the social systems already in place are fair and just.

The challenge of ethics education, therefore, becomes providing an environment that gives students experience with practicing ethical reasoning while simultaneously countering ideologies that portray engineering work as purely technical and apolitical. *Simulation* is one technique that can address both goals. In a framework illustrating ways that games may be used to teach ethics, Schrier (2015) suggests incorporating strategies such as role-taking and role-playing, storytelling, deliberation and discourse, collaboration, choices and consequences, application to real-world issues, and importantly, *simulation*. As she writes: “Games and simulations go beyond a story or case, however, because it can algorithmically incorporate many factors, and model an issue from many perspectives” (Schrier 2015, p. 412). By incorporating many factors and giving students a chance to experiment with outcomes, simulations can help to teach wise ethical practice. Simulation and gaming techniques have been found to be successful in teaching corporate social responsibility and business ethics (Bos et al. 2006). An educational simulation developed by Bos et al. (2006) gave business students practice at *perspective taking* to encourage students to understand the viewpoints of stakeholders outside of business environments, to foster awareness of cross-cultural issues in globalization, and to give students experience using moral reasoning.

Simulation and gaming have also been adopted for computer education, and specifically, for computer ethics education. For example, simulations have been used to teach programming (Galgouranas and Xinogalos 2018) as well as broader software engineering methods. Navarro and van der Hoek (2004, 2009) used simulation to teach software engineering management skills. Hof et al. (2017) used simulation to teach values such as collaboration and communication that are integral for Agile methods, a prevailing work process in software development. Fleischmann et al. (2011) used simulation as a core component of an information ethics course. They used cases derived from fieldwork in software ethics that asked students to play multiple roles and to collaborate on a decision. Their cases focused on nested decisions: a student playing one role would make an ethical decision that would then impact the next student’s choices and range of options. Fleischmann and colleagues’ simulation cases help students focus on personal moral qualities such as introspection, as well as understanding the ethical decision-making of others. Their project was successful in helping students realize the importance of their own, and others’, ethical decision-making. We expand on this work by using simulation to address two unmet challenges: (1) increasing students’ awareness of the relevance of ethical decision making to real-world technical work, and (2) practicing articulating ethical reasoning by working in a team to resolve ethical issues.

This paper describes how we designed the Privacy by Design (PbD) Simulation to engage students in an area of software development rife with ethical challenges: mobile application development. Mobile application developers face ethical challenges because they have considerable agency to make decisions about what data their applications collect (e.g. user location, photos, motion, sound), how long those data are kept, and whether they are shared or sold. The PbD Simulation presents participants with a sociotechnical task: writing a privacy policy for a mobile application and making technical changes to support that policy. As participants work together on the task, the simulation asks them to engage in work practices known to motivate ethical discussion called values levers (Shilton 2013; Shilton and Greene 2019). Values levers specific to mobile app developers include seeking app store approval, navigating policy constraints, navigating technical constraints, reviewing user requests, and interacting with third party data companies. Other values levers that apply to software development more broadly include leadership pressure and working on interdisciplinary teams.

**Fig. 1:**
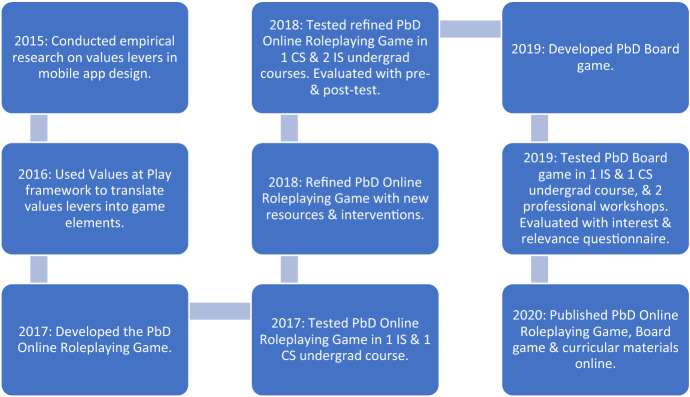
The PbD research and development process

Figure 1 illustrates the process by which we developed, refined, and evaluated two variations of the PbD Simulation: an online roleplaying game and a board game. Our paper proceeds as follows. First, we describe how we constructed the PbD Simulation to take advantage of simulation’s affordances for participant immersion and complex learning. We describes research into the real-world context of mobile application development that provided the scaffolding for our simulation design, and how we used the Values at Play (VAP) methodology (Flanagan and Nissenbaum 2014) to translate findings from this research into game design. We then detail the classroom tests of first an online roleplaying simulation and then a board game, and a series of evaluations of their success. Finally, we discuss how simulations such as the PbD Simulation can help address critical social challenges in engineering education.

## Simulation Development

To encourage ethical practice and illustrate debates found in real-world design settings, our simulation relies on *values levers*: practices found to encourage values discussions during technology development (Shilton 2013). For example, working across disciplinary barriers encourages teams to reflect upon their decision-making while explaining it to outsiders. Self-testing new systems helps developers experience sociotechnical possibilities and problems with their designs. Designing around both technical and policy constraints (such as what data can or cannot be sensed with available technology, or what is or isn’t allowed by a regulator) encourages team conversations about why the constraints exist and what values they support (Shilton 2013). Values levers suggest particularly effective entry points for values-oriented design in commercial technology development, and we designed our simulation to include these levers both to encourage ethical practice and to highlight how ethics discussions frequently arise within computing work.

To develop a simulation that would engage students in real-world activities that model how software developers encounter and debate ethics in professional settings, we took a two-step approach. First, we conducted qualitative research on ethical discussions in mobile application development to understand when and how values levers are deployed in practice. Then, we used the Values at Play approach (Flanagan and Nissenbaum 2014) to translate our observational findings into gaming elements within the simulation. The VAP Framework aids developers in creating games that engage individuals with values. It provides a systematic method for considering values during design, and incorporating those values into video games through three steps: discovery, translation and verification (Values at Play 2008). Table 1 summarizes each step in our VAP process, described below.

**Table 1:** Deploying the VAP framework

Discovery	Translation	Verification
Chose privacy as a key value Conducted field research to discover privacy values levers in mobile application development	Translated “privacy” into policy development task Online simulation: Translated values levers into roles, injects, and resources Board game: Translated values levers into roles, events, and resources	Piloted in a graduate seminar Deployed online simulation in five undergraduate courses Conducted a pre/post-test with undergraduate students in the same five courses Deployed board game in two courses and two professional workshops Conducted a survey evaluation of the board game player experience in two courses and one professional workshop

## Discovery

The VAP framework describes the discovery phase as: “locating the values relevant to a given project and defining those values within the context of the game” (Flanagan and Nissenbaum 2014). Game designers identify values and sources of values that influence the game. These include the key actors, values, ethical challenges, and any potential technical constraints (Flanagan and Nissenbaum 2014).

Our team chose to focus on *privacy* as a key value around which to build the simulation experience. Privacy is a frequent ethical challenge within mobile application development. In the US, there are few privacy standards with which mobile developers *must* comply.^2^ Instead, developers are able to make a range of decisions about what user data to collect and how to treat it, and must define for themselves what privacy means and to what degree they want to focus on this value. Complicating the discussion of privacy is the fact that the two major platforms for application development—iOS and Android—have different privacy policy requirements (Greene and Shilton 2018). Mobile developers often find themselves in the situation where they need a privacy policy, but are unsure what that policy should entail (Shilton and Greene 2019). We simulated this key decision-making situation to illustrate how ethics and policy questions can be entwined with technical work.

To find values levers related to privacy in mobile application design, two members of our team (Greene and Shilton 2018) conducted a critical discourse analysis of conversations about privacy in mobile development forums. Critical discourse analysis is a qualitative method for analyzing how individuals talk about and justify their practices (van Leeuwen 2008). We found that values reflection during application development is influenced by both the work practices of an individual or team and the politics and development culture of the larger platform ecosystem. Practices that opened values conversations included interacting with user analytics, as developers grappled with the (sometimes invasive) meaning of data about their users. Navigating platform approval processes was another lever for privacy conversations, as developers had to debate what kinds of data collection Apple or Google did or did not allow. Confronting technical constraints such as not being able to collect data continuously from phone cameras or microphones also spurred values conversations about why these constraints might exist.

As these examples suggest, analyzing privacy conversations in the mobile ecosystem illustrated the power of platforms to deploy values levers. Through both technical and policy means, Apple encourages frequent iOS developer conversations about privacy, while simultaneously enforcing narrow and problematic “notice and consent” privacy definitions. Google, on the other hand, exerts less overt technical and policy agency, and therefore developers engaged in less-frequent conversations about privacy. But Android developers responded to privacy problems with a wider and more creative range of solutions, because privacy requirements are not pre-defined by the platform (Greene and Shilton 2018). Based on this research, our simulation models both the politics and development culture of a platform ecosystem and the work practices of the team.

### Translation

Translation is the process of developing game play elements that raise, enact, or help students question values within the game (Flanagan and Nissenbaum 2014). Our translation process focused on constructing simulation elements that would encourage participants to particularize and make decisions about the ethical challenge of what user data to collect.

First, we translated the ethical challenge into an online roleplaying simulation. We created a scenario in which participants are members of a fictional health application development team. The team is charged with porting an existing application from the permissive “Robot” platform (modeled after Android) to the more restrictive “Fruit” platform (modeled after iOS). Participants were tasked with creating two outputs describing a set of (1) policy changes and (2) associated technical changes needed for the transition. This set-up evoked the privacy decisions that real-world developers must make when moving their product between platforms, and also engaged the tensions between the two platforms and their differing privacy policies that we observed in our observational research.

We also assigned participants contrasting team roles, such as the project manager, software developer, or user experience developer, to experiment with team diversity, which had been shown be an important values lever in previous research (Shilton 2013). Participants received short descriptions of their roles as well as subtle hints (shown in bold below) about what that role might particularly value. The Software Manager is told to “lead the team to *successful completion* of a software project.” The Software Developer “collaborates on the designs and development of *technical solutions*.” Finally, the User Experience Designer “*advocates for the user* during the design and development of projects. By giving each role slightly different priorities we hoped to seed explicit values conversations.

Next, we created *injects*—messages from non-player characters that would be deployed throughout the online simulation—based on factors found in our empirical research. An inject from a fictional friend introduces possible policy constraints by emailing a blog article about HIPAA to participants. An inject from a fictional marketing director introduces third-party data access by asking participants to consider allowing partnership—and user data sharing—with an insurance company. We also experimented with the impact of leader advocacy, an important lever for encouraging values conversations in earlier research (Shilton 2013), by having the head of the legal department express concerns about data breaches.

Finally, we used real-world developer privacy discussions as resources for student participants. Students were directed to forum discussions where software developers had negotiated consensus on the meaning of privacy. We also gave participants other resources to guide the online simulation: a design document specifying the current workings of the app, including how and when it collects personal data; and the “Robot” and “Fruit” application store policy guidelines.

After developing the scenario, roles, injects, and resources, we brought the online roleplaying simulation to life using the ICONS platform (https://www.icons.umd.edu/): a web-based environment that facilitates roleplaying, discussion and deliberation, and decision-making processes. Students had individual ICONS accounts, and when they logged in, were given a role and assigned to a team. A welcome email from a fictional manager described the online simulation task, and students could email each other within the platform to discuss the assignment and their goals. The students could also author proposals on the platform (in this case, describing both policy changes and technical changes), and could vote on others’ proposals. Injects appeared as emails from fictional characters alongside students’ email communication with their team. Table 2 summarizes values levers we found in the empirical research and how we translated them into simulation elements.

**Table 2:** Translation from values lever to simulation element

Values lever	Simulation element
Navigating platform approval process	*Task:* porting the app from the “Robot” to the “Fruit” platform. *Resource*: “Robot” and “Fruit” policy guidelines
Confronting policy constraints	*Inject:* Email from a fictional friend bringing up HIPAA policy constraints
Confronting third party data uses	*Inject:* Email from marketing director asks participants to consider allowing user data sharing with an insurance company
Team diversity	*Resource:* Contrasting team roles
Leader advocacy	*Inject:* Email from the head of the legal department expressing concerns about data breaches
Peer influence	*Resource:* forum discussions where software developers negotiated consensus on the meaning of privacy

### Iteration: From Simulation to Board Game

The ICONS platform provided a rich roleplaying environment, but it is also labor- and cost-intensive to run. Participation requires contracting with ICONS and paying a fee that supports the platform, setting up individual accounts for students, and participating in days of online interactions overseen by a moderator. After our positive experience running the online simulation, we wanted to develop a less labor-intensive and free way for educators around the country to use the experience in their classrooms. Our team therefore developed a board game version of the PbD Simulation, which is freely downloadable https://evidlab.umd.edu/privacy-by-design-the-game/ and can be played in a single class session. The game scenario mimics the original online roleplaying simulation: a team must create a new privacy policy for the “Fruit OS” store. The team plays cooperatively. Each member draws a card that assigns them to one of the same roles assigned in the online simulation (see Fig. 1). The layout of the game board then guides a series of privacy decisions: what kinds of data to collect, and who to share that data with (see Fig. 1). Participants make each privacy decision as a team, and either gain or lose two types of *resources* for each decision: “developer time” and “user trust.” Developer time represents the money, labor, and resources necessary to build and maintain applications. Developer time is important because it helps the team build more, bigger, and better products. User trust represents the trust of the customer base in the application and company. User trust is important because it helps ensure customers want to use the application. Develop time and user trust combine to determine the game score; if either resource runs out, the team loses the game. Although the team works cooperatively to make decisions, each assigned role is given secret, conflicting objectives that advise each participant to monitor a particular resource and ensure a certain level of that resource is maintained throughout gameplay.

**Fig. 2:**
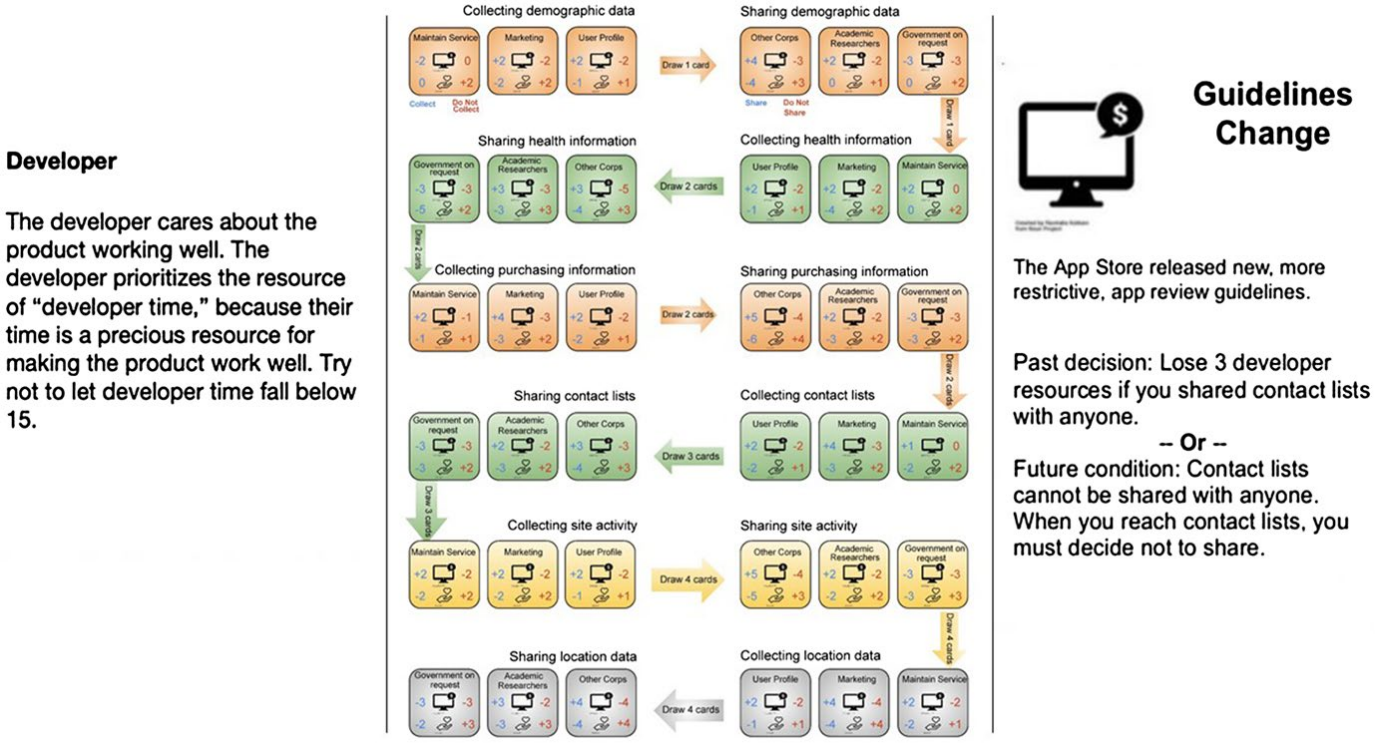
Role cards, gameboard, and an event card

The online roleplaying simulation’s injects are replaced by event cards, which are drawn after every set of privacy decisions. Event cards incorporate values levers discovered during the earlier fieldwork-based discovery phase of the project phase, including interacting with app store guidelines (see Fig. 2), receiving feedback from users, or following changes in law. Event cards mimic actual events observed during the earlier fieldwork.

## Verification: Evaluating the Online Simulation and the Board Game

In the VAP Framework, verification is the name given to the evaluation phase: ensuring that the intended values are conveyed through the playing of the game and understanding the impact of the game on players. We tested the initial version of the online roleplaying simulation in 2017 in two undergraduate courses: Database Design and Modeling (undergraduate, 50 students, classroom) and Programming Handheld Systems (undergraduate, 88 students, classroom). After the pilot testing in two courses, we made modifications to scenario content, policy resources, and intervention materials. We also designed an introductory video featuring actors playing various online simulation characters to present the fictionalized storyline and improve student immersion in the activity.

We then ran the online roleplaying simulation in three additional courses in 2018: Introduction to Programming for Information Science (undergraduate, 108 students, classroom), Programming Handheld Systems (undergraduate, 145 students, classroom), and Teams and Organizations (undergraduate 48 students, classroom). The online simulation was run in three 50-min sessions or one 75-min session (depending on class format) and included a debrief at the end. Participants who agreed to participate in the research portion of the project (approved by our university Institutional Review Board) were also given the pre- and post-test (discussed in detail below).

Board game play requires one 75-min session and included a debrief at the end and surveys to evaluate student’s and professional’s game-playing experience. We played the board game in two courses in 2019: Introduction to Information Science (undergraduate information science, 127 students, classroom), and Programming Handheld Systems (undergraduate computer science, 155 students, classroom). We also played the board game in a workshop at the 2019 Android Summit conference (professionals, 15 participants, office space), and an Amazon Web Services User Group Meet up (professionals, 34, office space).

Our pedagogical goal was for participants to gain experience recognizing, particularizing, and making decisions about the ethical issues entangled within technology design. Our original plan was to evaluate the online roleplaying simulation and the board game’s success through a pre- and post-test taken by participants. After reviewing standard measures such as the Defining Issues Test (DIT) (Rest et al. 1999) and the Engineering and Science Issues Test (Borenstein et al. 2010), we determined that the moral maturity models used in those measures were not a good fit for our intervention. Our study does not span long periods of time and Borenstein and colleagues did not find significant results when running an intervention-based study. Therefore, we decided to design our pre-/post-test instrument to measure ethical sensitivity, the combination of moral imagination and ability to identify ethical issues (Clarkeburn 2002). Though we considered using the Test of Ethical Sensitivity in Science and Engineering (Borenstein et al. 2008), we ultimately decided that tailoring the instrument to the specific privacy and computing issues that the online simulation and board game covered would be the most likely to provide a valid measure of the effects of the intervention and anticipated changes in developers’ ethical thinking.

Our pre/post-test, modeled after the Defining Issues Test, presented students with three scenarios about mobile development. The first featured a story about a developer grappling with decisions about user data tracking. The second discussed a developer who had discovered a bug in their company’s code. And the third focused on a developer’s decisions about user data sharing after receiving negative user feedback. Students were then asked whether a series of given choices were “ethical issues in the story” (a standard indicator of ethical recognition). The choices contained relevant ethical issues (e.g. protecting users, deciding between company needs and user values) as well as non-ethical issues (e.g. delegation of work tasks among job responsibilities).

However, even our adapted measure of moral sensitivity proved problematic.^3^ In our analysis, we found that students scored so highly on the *pretest* that measuring change on the *posttest* was impossible. Perhaps because issues of mobile privacy and engineering responsibility have been in the news, or perhaps because of exposure in other courses, students recognized the ethical issues at play even before engaging in our online simulation. From this, we concluded that undergraduates in both our computer and information science programs were already skilled at *identifying* ethical issues in computing. While this is good news for moral sensitivity in technology design generally, it complicated our evaluation plans.

What the DIT does not measure are components of ethical sensitivity beyond identification, such as particularization (understanding the problem) and judgment (making decisions). The PbD simulation encourages students to engage in both particularization and judgment. And the DIT is also not responsive to our goal of highlighting ethical debate and decision-making as relevant to technical education. As a result, we decided on a new evaluation technique. We borrowed an evaluation strategy from another area of computing ethics: evaluating students’ experience with an activity rather than their moral learning (Grosz et al. 2019). Inspired by the “Embedded EthiCS” program at Harvard University, which similarly attempts to demonstrate the entanglement of social and technical topics in computer science (although not explicitly through gameplay), we adapted a five-question survey used by Barbara Grosz and colleagues. We asked students in two undergraduate course sections of Introduction to Information Science and one section of Programming Handheld Systems whether they found the activity interesting, but also, whether they found it *relevant*, with the idea that this would help us understand if we had succeeded at illustrating that ethical decisions are part and parcel of technical work. We also asked whether the game helped the students to think more about the ethical issues and decisions; whether the game increased their interest in learning about ethical issues and decisions in app design; and whether students were interested in learning more about ethical implications in technology.

Roughly 2/3 of the 224 student respondents agreed that they found the game interesting (65.2%) and that it helped them think more about ethical issues 65.5%. Over half agreed that the game was relevant (56.2%) and increased their interest in ethical issues (54.4%). The students also overwhelmingly expressed interest in learning more about ethics in technical work in class (90.1%) and outside of class in workshops or seminars (82.1%). There were also significant differences between the courses on responses to whether they found the game interesting and relevant. A two sample *t* test found that students in the mobile development course (n = 124) (Programming Handheld Systems) found the game both more interesting (mean = 3.89 vs. 3.47 on a 5 point Likert scale; *p* < .001) and more relevant (mean = 3.76 vs. 3.31; *p* < .0001) than students in the general Introduction to Information Science (n = 100). This indicates that the game’s focus on ethical issues in mobile development is a better fit for a technical course that focuses on mobile development. Though a small sample of industry participants responded to our evaluation (n = 8), 87% agreed that the game was interesting and 62% agreed it was relevant. These numbers indicate that interest in such a game may increase as participants increase their experience with real-world mobile development.

## Conclusion

The evaluations raised important questions for simulation-based ethics education moving forward. The finding that interest and engagement with the PbD simulations was higher for students in topically-focused courses indicates the importance of adapting ethics education tools to the entwined ethical and technical quandaries of particular design areas. Illustrating ways in which ethics is directly relevant to technical work is critical to ethics education. In response, we are working on ways to adapt the mechanisms of the PbD Simulations for other computing ethics topics. Currently, we are working with experts in content moderation to develop a game with similar mechanics to help students consider the challenging social and technical decisions inherent in AI-assisted online content moderation. We hope to provide educators with both powerful games and the means to shape their own games to suit their course content. Development of a wide variety of simulation activities tailored for diverse computer ethics issues can further both education and evaluation efforts.

In addition, our field continues to struggle with identifying and measuring effects of ethics education interventions. Our team has plans for ongoing evaluations to compare the game with other interventions focused on ethical learning in computer science. For example, using qualitative observation to compare either (or both) Pbd Simulations with legacy methods such as case studies, as well as emerging methods such as design fictions (Wong et al. 2018), can help us understand whether the game increases students’ reflexivity, the diversity of the ethical issues they consider, or the wisdom of their decisions.

Software engineers are facing international scrutiny for unethical behavior. Even celebrities are getting in on the act. In a tweet stream, actor Nanjiani (2017), famous for playing a software developer on TV, expressed:

I know there’s a lot of scary stuff in the world [right now], but this is something I’ve been thinking about that I can’t get out of my head. As a cast member on a show about tech, our job entails visiting tech companies/conferences etc. We meet [people] eager to show off new tech. Often we’ll see tech that is scary. I don’t mean weapons etc. I mean altering video, tech that violates privacy, stuff [with obvious] ethical issues. And we’ll bring up our concerns to them. We are realizing that ZERO consideration seems to be given to the ethical implications of tech. They don’t even have a pat rehearsed answer. They are shocked at being asked. Which means nobody is asking those questions. … Only “Can we do this?” Never “should we do this? We’ve seen that same blasé attitude in how Twitter or Facebook deal [with] abuse/fake news.

The reasons that developers too infrequently ask “should we do this?” are complex, ranging from challenges in teaching ethics to fundamental challenges in the culture of engineering education. Neither can be completely solved by one educational intervention, but we believe that simulation can make an appreciable difference. An experiential learning approach through simulation not only allows software engineering students to practice ethical wisdom, but also directly incorporates ethical decision-making as a component of technical work.

## Footnotes

1 See, for example, the crowdsourced list of tech ethics courses maintained by Casey Fiesler: https://docs.google.com/spreadsheets/d/1jWIrA8jHz5fYAW4h9CkUD8gKS5V98PDJDymRf8d9vKI/edit#gid=0.

2 This may be changing due to new legislation in the state of California, although it will be some time before it becomes clear how mobile developers will interact with new data privacy laws.

3 We are not alone in struggling to document and quantify moral learning. There is a long history of scholarship devoted to the problem of evaluation in ethics education (Elliott and Stern 1996), and even an unusual half-hour of American network television that illustrates the difficulty of trying to quantify moral learning (Bell 2019).
